# Modeling of Polymer Composites and Nanocomposites

**DOI:** 10.3390/polym17141944

**Published:** 2025-07-16

**Authors:** Rafał Grzejda

**Affiliations:** Faculty of Mechanical Engineering and Mechatronics, West Pomeranian University of Technology in Szczecin, 19 Piastow Ave., 70-310 Szczecin, Poland; rafal.grzejda@zut.edu.pl

## 1. Introduction

The importance of polymer composites (PCs) and nanocomposites (PNs) has increased significantly in recent years due to their enhanced material performance, sustainability and versatile applications [1,2,3,4,5]. These composites are constructed by combining a polymer matrix, reinforcement (e.g., fibers) and filler, most commonly either by introducing a monomer into the system and polymerizing it, or by directly introducing the polymer into the system [6,7]. Natural polymers are also used as a matrix for composites [8]. In addition, natural fibers are applied as reinforcements in PCs as an alternative to synthetic fibers, particularly in terms of improving the mechanical properties of the matrix [9,10,11]. Furthermore, natural fiber-reinforced composites (FRCs) show exceptional economic efficiency and are environmentally friendly, making them highly suitable for a wide range of applications [12,13,14].

Recent advancements in the field of PCs and PNs have focused on a number of aspects, including processing techniques, product innovations, surface and color treatments and the investigation of critical properties [15,16,17]. Moreover, research is being carried out on layered hybrid composites with bio-based raw materials in order to provide sustainable alternatives in structural applications with an emphasis on diversifying the sourcing of raw materials [18,19]. To optimize functional characteristics, specific additives are introduced into the composite formulation, which play a key role in enhancing interfacial adhesion, mechanical strength, dimensional stability and resistance to environmental degradation, thus increasing the overall durability and functionality of the material [20,21,22,23,24].

Composite products find a wide range of applications in the automotive, transport, sports, shipbuilding, packaging, medical and engineering industries, among others [25,26]. Advances in polymer composite technology continue to lead to improvements in mechanical performance, biodegradability and recyclability [27,28]. Nanotechnology and fiber modification techniques are being explored to further enhance the strength, thermal stability and fire resistance of composites. All this underlines how important PCs and PNs are today and that their modeling and further extensive research on them is needed. Some of this research is presented in the Special Issue of *Polymers* entitled “Modeling of Polymer Composites and Nanocomposites”, the introduction to which is this Editorial.

## 2. Overview of Papers Published in the Special Issue

Umer et al. (Contribution No. 1) documented the feasibility of converting clean and renewable energy sources into electricity through the use of piezoelectric materials. They used a micromechanical study of the effect of adding silica nanoparticles to a polyimide matrix on the piezoelectric–elastic response of piezoelectric fiber-reinforced nanocomposites [29]. It was found that the addition of silica nanoparticles to the polymer improves the elastic and piezoelectric properties of the piezoelectric fiber nanocomposites. At the fiber volume fraction of 60%, the nanocomposite with polyimide filled with 3% silica exhibits a 39% improvement in the transverse Young’s modulus, which is almost a 32% improvement in transverse shear modulus and 37% improvement in the piezoelectric coefficient compared to the composite without nanoparticles. It was also shown that the piezoelectric–elastic properties, described by the above-mentioned piezoelectric constants, can be enhanced with a reduction in the diameter of the nanoparticles.

The second paper by Umer et al. (Contribution No. 2) deals with multifunctional PCs containing micro/nano hybrid reinforcements, which are currently attracting intense attention in the field of materials science and engineering. In the paper, a multi-phase analytical model was developed to study the effective electrical conductivity [30] of PCs with silicon carbide microcarriers and carbon nanoparticles. Before the percolation threshold, it was observed that the addition of nanoparticles with uniform dispersion can improve the electrical conductivity of the PCs tested. It was also shown that the electrical conductivity increases more by reducing the size of the nanoparticles.

Issues related to carbon fiber-reinforced polymer concrete beams for safety design are addressed in the paper by Maidi et al. (Contribution No. 3). It presents the structural response of such beams integrated with rigid frames during seismic events. Multi-scale simulations and parametric analyses were performed to optimize the residual state and global performance. The main parameters considered were tensile and compressive reinforcement, concrete strength, height-to-width ratio, cross-sectional coverage and confinement level, all of which are important for understanding their impact on seismic performance [31]. The results of the parametric analysis highlight the increased ductility and higher load carrying capacity of the tested reinforced beams compared to reinforced concrete beams [32]. This indicates that it is possible to design carbon fiber-reinforced polymer concrete components that could enhance ductile frames with increased energy dissipation and be suitable for use in non-corrosive seismic-resistant buildings.

Marashdeh and Madkhali (Contribution No. 4) considered heavy metal doping as a means of enhancing the radiation protection efficiency of *Nigella sativa* eumelanin polymers. The herbal polymer was doped with iron (Fe), copper (Cu) and zinc (Zn) and it was examined how this doping affects the gamma ray shielding properties of the polymer. The doping of eumelanin polymers with the aforementioned components has been shown to be particularly effective in enhancing gamma ray protection at low energies, with Cu providing the most significant overall improvement, making these composites suitable for applications requiring enhanced radiation protection at lower gamma ray energies [33,34].

To improve the thermal and electrical properties of organic electronics, one potential research area is the study of composite layers that combine semiconducting organic polymers with inorganic nanoparticles, namely gold nanoparticles (AuNPs). One such promising composite system is based on two semiconducting polymers, PDPP4T and PCPDTBT, which are known for their small optical band gaps and high charge carrier mobility, making them ideal candidates for organic solar cells [35]. The inclusion of AuNPs in the blends of these polymers offers a unique opportunity to explore how nanoparticles affect the thermal transformations in these materials, ultimately affecting material properties and device performance. With this in mind, Jarka et al. (Contribution No. 5) used variable temperature ellipsometry [36] to examine thermal transitions in PDPP4T/PCPDTBT/AuNPs composite layers. They made their conclusions based on the developed phase diagram [37] for the PDPP4T/PCPDTBT blend using variable temperature spectroscopic ellipsometry with differential scanning calorimetry serving as a reference technique. The incorporation of AuNPs was evaluated to significantly affect the thermal stability and crystallization of the material, which is essential for organic field-effect transistor applications.

Understanding the structural dynamics of semiflexible polymers in an implicit solvent under different conditions provides valuable insights into their behavior in diverse environments. For this reason, Williams and Gray (Contribution No. 6) investigated the effect of the angular width of the bending potential on structural state behavior and conformational variation using microcanonical analysis [38]. A range of angular widths was considered, with the widest value corresponding directly to the classical semiflexible polymer model [39]. It was shown that as angular width decreases, structural variability within states decreases, structural state overlap decreases and conformations become more stable, leading to an expansion of the parameter space dominated by individual structures.

It is well known that adding carbon and glass fibers to polymer matrices improves their properties such as strength, stiffness and impact resistance. However, most of the existing research on the effects of these additives mostly concerns the study of single factors—such as fiber orientation, filling pattern or interlayer adhesion—without fully considering the interaction of these parameters in practical load-bearing applications. Furthermore, the intrinsic anisotropy of parts produced by Fused Deposition Modeling (FDM) [40], compounded by inconsistencies in layer bonding and fiber deposition, increases the complexity of predicting their mechanical properties. To address these challenges, Nemes et al. (Contribution No. 7) conducted a comprehensive, full-field deformation and damage analysis of carbon and glass fiber-reinforced 3D printing filaments based on Polylactic Acid (PLA) and Polyethylene Terephthalate Glycol (PETG) [41]. The effects of printing parameters—including infill pattern, density and structure orientation—were investigated under both bending and compressive loading. The study found that although carbon and glass composites provide a more stable structure, their mechanical properties are generally inferior to PLA and PETG. These materials are less sensitive to changes in fill pattern and fill factor, so their strength is more uniform across different printing settings. Tests showed that the carbon and glass fiber PLA filaments behaved similarly, but in terms of both tolerable force and achievable deformation, the carbon fiber PLA filament performed slightly better. If bending is the expected stress for the structure, it is better to use a basic PLA material, which showed better results than fiber-reinforced filaments. Carbon fiber PLA and glass fiber PLA filaments have also been shown to perform better with mesh infill patterns and lower infill densities. This can be of practical importance when a low infill density has to be chosen (to optimize weight) [42] and when bending stresses are expected. According to tests, the optimum infill value for these materials is between 20 and 30%, so it was suggested that for load-bearing components there is no reason to use a higher infill.

The growing demand for fully recyclable composites has spurred extensive research into thermoplastics, valued for their recyclability and excellent mechanical properties. Recently, thermoplastic polymer blends have gained attention for their enhanced recyclability and sustainability, as well as their ability to improve thermal stability, viscosity and manufacturability. However, limited data are available on the mechanical characterization of composites that incorporate these blends, particularly for thermoplastic recycling. To expand on this knowledge, Hao et al. (Contribution No. 8) investigated the stress–strain behavior of the following three polymer blends relevant to structural applications: PES/PEEK, PPS/PEEK and HDPE/PP. They then performed numerical analysis to predict the mechanical properties of unidirectional FRCs using each of these blends as a matrix. Representative Volume Element-based simulations [43,44] were used for this purpose. Finally, the suitability of the blends tested to produce fully matrix-recycled composites was critically evaluated. This has provided valuable preliminary insight into the mechanical viability and sustainability benefits of using recycled thermoplastic blends as matrices for unidirectional FRCs.

The effect of resin composition on the photopolymerization of zirconia ceramics produced by digital light processing [45] using additive manufacturing (AM) was studied by Kuang et al. (Contribution No. 9). An oligomer was considered as an additive to the photosensitive resin. The dependence of the content of this additive on the viscosity and curing properties of ceramic suspensions was investigated. The results demonstrated that the introduction of oligomers is conducive to enhancing the cross-linking density and reducing defects. Furthermore, zirconia ceramics fabricated by photopolymerization with oligomer photosensitive resin have been shown to exhibit excellent mechanical properties, greatly expanding the potential applications of high-performance zirconia ceramic components using AM.

Rachtanapun et al. (Contribution No. 10) successfully developed antistatic and anti-flammable biodegradable PCs by melt-blending polybutylene succinate with epoxy resin, polybutylene adipate-co-terephthalate and MgO particles. The addition of MgO improved the thermal decomposition behavior and water resistance of the blends, which could be attributed to the high thermal stability and hydrophobicity of the metal particles. The produced PCs showed an improvement in the V-1 rating [46] of flame retardancy, indicating an enhancement in the flame retardancy of biodegradable composite films. MgO served as a flame retardant, increasing the strength of the residual charcoal from the cross-linked matrix. The antistatic properties of the composites were improved using both plasma technology and the sparking process [47]. The antistatic effect of the plasma sputtering process was found to be better than that of the sparking process, as the sputtering method increased the uniform dispersion of metal nanoparticles on the composite surface. The resulting antistatic and anti-flammable biodegradable PCs with improved properties have potential for use in packaging, electronics and automotive applications.

## 3. Conclusions

The collective contributions to this Special Issue reflect a comprehensive and future-oriented exploration of sustainable practices and innovative technologies in the field of polymer composite and nanocomposite materials and products. The following conclusions can be drawn from the content presented in the papers covered in this Special Issue:

1. The introduction of suitable nanoparticles or dopants into polymer composites leads to improved piezoelectric properties and increased electrical conductivity efficiency, but also, for example, to effectively increase protection against gamma radiation at low energies.
2. The application of carbon fiber-reinforced polymer concrete offers hope for the possibility of designing ductile frames with enhanced energy dissipation, suitable for use in non-corrosive seismic-resistant buildings.
3. Adding carbon and glass fibers to polymer matrices used in 3D printing can improve their properties such as strength, stiffness and impact resistance.
4. Thermoplastic polymer blends are gaining attention for their increased recyclability and sustainability, as well as their ability to improve thermal stability or even flame retardancy and broad manufacturing capabilities.

## Abbreviations

The following abbreviations are used in this paper:

AM	Additive manufacturing
AuNPs	Gold nanoparticles
FDM	Fused Deposition Modeling
FRCs	Fiber-reinforced composites
PCs	Polymer composites
PETG	Polyethylene Terephthalate Glycol
PLA	Polylactic Acid
PNs	Polymer nanocomposites
